# COVID-19: A Therapeutic Approach Based on Pathophysiological Staging

**DOI:** 10.2174/1874306402014010032

**Published:** 2020-10-13

**Authors:** Christian Domingo Ribas

**Affiliations:** 1 Pulmonary Service, Corporació Sanitària Parc Taulí, Barcelona, Spain; 2 Departament of Medicine, Universitat Autònoma de Barcelona (UAB) Barcelona, Spain

## Abstract

The COVID-19 pandemic is a recently emerging problem. This has caused that the knowledge of the disease has been progressive and, therefore, the therapeutic decisions have been conditioned by this lack of knowledge on the one hand and by the therapeutic limitations on the other. Many published studies are methodologically weak and their conclusions, of limited value, have contributed to creating confusion on the therapeutic approach of the disease.

In the present paper, we propose a therapeutic approach based on a new disease staging. The therapeutic approach is divided into two big sections: the pharmacological treatment for the phase of viral replication, cytokine storm or late respiratory events (which includes the adult respiratory distress syndrome (ARDS)) and the treatment of the respiratory failure In every stage, we discuss the pathophysiology and comment (accept or rule out) the pharmacological options according to the present evidence. Moreover, we indicate how respiratory failure should be treated. Some characteristics are based on the evidence found in the literature. Others are the result of my experience in other situations.

## 1 INTRODUCTION

During the last five months, a new disease has appeared on the medical horizon: the COVID-19 infection. Initially, in response to the first reports from China, COVID-19 was considered a mild disease with high infectivity (being termed the “new influenza”). Sooner than expected, the infection moved to Europe, hitting Italy particularly hard. At that moment, it became evident that the disease had many different faces and that its severity was due to respiratory failure. At my hospital, we saw more than 2700 patients with COVID-19 infection, more than a thousand of whom needed hospital admission. In this paper, I summarize my experience acquired during the worst moments of the pandemic, present an interpretation of the literature, and outline my views on the pathophysiology of the disease.

A first attempt to classify the different stages of the disease was made by Siddiqi *et al*. [1]. For these authors, the pulmonary phase occurs before the hyperinflammation phase, without any overlap between them. In stage I, the constitutional symptoms are mild rather than moderate or severe. In stage II, it is difficult to explain how patients without hypoxia (stage IIA) could be short of breath. The definition of stage IIB is PaO2/FiO2 < 300, which corresponds to diffuse alveolar damage, and may precede the development of Acute Respiratory Distress Syndrome (ARDS). It is difficult to understand how an episode of ARDS might occur during a phase of hyperinflammation, since ARDS is the consequence rather than the cause.

### 1.1 New Pathophysiological Classification Proposal and Therapeutic Approach

**Fig. (1):**
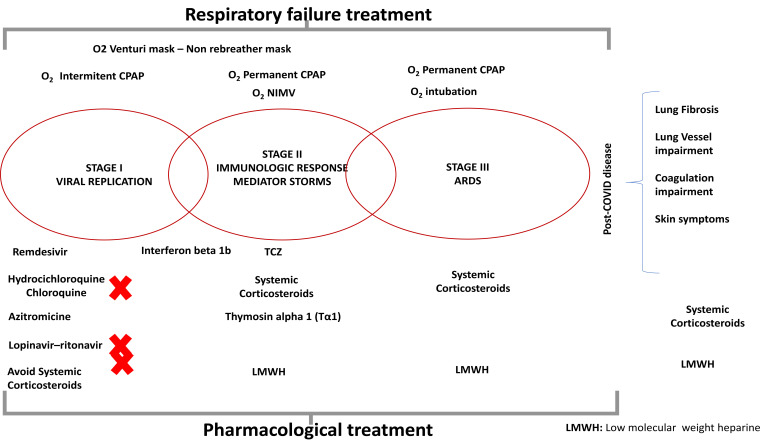
LMWH: Low Molecular Weight Heparin.The red crosses show the drugs without evidence of success for COVID – 19 infection.

We propose a different pathophysiological classification basically driven by respiratory involvement, which divides the disease into four stages (stages I, II, and III, and post-COVID complications) (Fig. **1**). Stage I corresponds to the viral replication phase, in which constitutional symptoms are mild or absent. As the disease progresses, as a consequence of the activation of macrophages and other cells, there is an overproduction of early response proinflammatory cytokines (tumour necrosis factor [TNF], IL-6, and IL-1β). In some cases, this initially protective immunological response occurs in an exaggerated manner, resulting in what has been described as a “cytokine storm” (stage II). This can cause severe constitutional symptoms (not represented in the figure) and/or progress to cause lung damage (pneumonia). As the disease worsens, the hypoxia may eventually evolve into a classic ARDS with early lung fibrosis related to the SARS-CoV-2 infection itself; platelet-fibrin thrombi may appear in small arterial vessels (<1mm in diameter) causing hypoxemia and reducing CO transfer capacity [2]. In what follows, the scaling of the pharmacological treatment options and the oxygenation techniques available (and appropriate) will be discussed in accordance with this staging.

### 1.2 Pharmacological Treatment

During viral replication, Chloroquine (CQ) and the more potent Hydroxychloroquine (HCQ) have shown some in vitro activity against SARS-CoV-2 [3, 4]. In a retrospective study, these drugs were significantly associated with viral load reduction in COVID-19 patients, and their effect was reinforced by azithromycin [5]. Higher CQ dosage is not recommended for critically ill patients with COVID-19, and in general clinical trials have provided inconsistent messages. A recent study concluding that these drugs were not effective against COVID-19 was withdrawn from publication due to methodological issues. Given that the study was deemed unreliable, one could easily be deceived into thinking that its conclusion was incorrect, and that these drugs should in fact be recommended for treatment or prevention [6]. This is a misapprehension: the evidence favouring the recommendation of CQ and HCQ is still uncertain. Azithromycin alone has not been tested, and the association of lopinavir–ritonavir, which has a high number of side effects, has not shown any benefits [7]. The initial information on remdesivir was confusing since a randomized, double-blind, placebo-controlled, multicentre trial in China [8] was inconclusive, although the study did not reach the sample size calculated. A later study [9] showed that remdesivir offered a 31% faster time to recovery than placebo (*P*< 0.001) and a lower median time to recovery (11 days *vs* 15), and reduced the mortality rate from 11.6% to 8.0% (*P* = 0.059). Corticosteroids are unlikely to be used since they would favour viral replication.

As a result, at the present time, remdesivir seems to be the only treatment recommended for stage I patients, and therefore the only drug recommended for treating viral replication [10].

Stage II is the storm mediator phase, with increased plasma concentrations of proinflammatory cytokines [11]. Moreover, histopathological examination of lung tissue from deceased patients with COVID-19 showed evidence of extensive alveolar oedema, proteinaceous exudate and patchy inflammatory cellular infiltration [12]. These findings suggest that severe SARS-CoV-2 infection is associated with a cytokine storm and pulmonary inflammation secondary to a dysregulated host immune response. IL-6 is one of the most important mediators that have been found to increase. Autopsy reports from SARS patients showed high levels of inflammatory cytokines in cells expressing angiotensin-converting enzyme 2 [13], the functional receptor for SARS-CoV and with an even higher affinity for SARS-CoV2 [14]. Immunologically, C-Reactive Protein (CRP) and IL-6 are closely intertwined. IL-6 is known to induce gene expression and the release of CRP from the liver. In a paper including a pilot study that allowed the calculation of the sample size for a subsequent prospective study, peak levels of IL-6 followed by CRP were highly predictive of the need for mechanical ventilation. This suggests the possibility of using IL-6 or CRP levels to guide the escalation of treatment in patients with COVID-19-related hyperinflammatory syndrome [15].

Tocilizumab (TCZ), a blocker of the soluble and membrane receptors for IL-6, has been shown to be effective in patients with severe disease in two retrospective studies [12, 16]. TCZ is indicated for several diseases including rheumatoid arthritis. Normally patients receive one dose per month. In COVID-19 patients, up to three doses in 24 hours have been proposed. Although there is no clinical evidence to support this, from a pathophysiological point of view the proposal makes sense: in cases of molecules acting through membrane receptors, the increase in the blood mediator causes an externalization of cell receptors. Given that TCZ is a receptor blocker, it may cause a transient increase in membrane receptors due to the persistence of high IL-6 concentration in blood. Moreover, Luo P. *et al*. [16] observed that the serum IL-6 levels tended to spike at first and then decrease after TCZ therapy in 10 patients. This transient increase could also be explained by the theory just mentioned. As a result, since IL-6 release to the blood is very intense, the administration of more than one isolated dose of TCZ would be acceptable in the absence of other clinical information. Incidentally, TCZ is very well tolerated.

Other mediators released during COVID-19 disease such as interferon β-1 (17) or thymosin alpha 1 (18) seem to offer a beneficial effect and their low concentration can be harmful. In a multicentre, prospective, open-label, randomized phase 2 trial in adults with COVID-19, interferon β-1 reduced the median time from the start of treatment to a negative nasopharyngeal swab (the primary endpoint) from 12 days to seven in the treatment group, and the median hospital stay from 14.5 days to 9 [17]. Lymphocytes play an essential role in fighting against viral infections; therefore, boosting the number and enhancing the antiviral function of T cells in COVID-19 patients is of a paramount value for successful recovery. Lymphocytopenia and T-cell exhaustion are common in acute COVID19 patients. Thymosin-α1 (Tα1), a polypeptide hormone produced by thymic epithelial cells, can effectively increase T-cell numbers, support T-cell differentiation, maturation, and reduce cell apoptosis. Tα1 has been successfully used in clinical practice to treat patients infected with hepatitis B (HBV), hepatitis C (HCV) and Human Immunodeficiency Viruses (HIV). In a retrospective study, it reduced mortality in severe COVID-19 patients (11% **vs**. 30%, p=0.044), enhanced blood T-cell numbers in COVID-19 patients with severe lymphocytopenia and decreased the levels of T-cell exhaustion markers in CD8+ T cells [18]. IL-1 has also been involved in the cytokine storm in sepsis and COVID-19 disease [19]. Anakinra, an IL-1 receptor inhibitor has also been postulated as an alternative although the evidence of its clinical effectiveness is yet limited [20].

The controversy surrounding the benefits of corticosteroids seems to be reaching its end. Barely discussed in ARDS, in the hyperinflammation stage II a limited burst of 0.5-1 mg/kg of methylprednisolone in two intravenous doses for three days [21] reduced severity, mechanical ventilation requirement and mortality. More recently, low doses of dexamethasone seemed to offer similar benefits in ventilated patients [22]. Other blockers of mediators, such as acalabrutinib [23], a selective Bruton tyrosin kinase inhibitor with an interesting theoretical basis, or statins [24], widely used as anti-inflammatory drugs, need to be clinically tested. Among these drugs, there is no clear evidence to decide which one to test first, although TCZ seems to be the most readily available and probably should be the first to be used. Combinations with other alternatives such as corticosteroids or other monoclonal antibodies should be considered.

In stage III, the therapeutic options are limited. Corticosteroid use in classic ARDS has been under discussion for the last 40 years. Some ventilated patients may present benefits [22]. Maintenance treatment (including nutrition) seems to be crucial. In stages II and III, low molecular weight heparin (LMWH) has been included. The recently published ACCP guidelines [25] are very conservative, considering that information on coagulation (D-dimer and partial thromboplastin time) and inflammatory markers (ferritin, LDH, C-reactive protein) is easy to obtain and D-dimer has been found to correlate with in-hospital death in a multivariate analysis [26]. Certain authors [27] (and I myself) tend towards a more aggressive attitude. Prophylactic doses of LMWH are recommended for hospitalized patients with COVID-19 to prevent venous thromboembolism and medium or treatment doses of LMWH should be considered for those with significantly raised d-dimer concentrations, due to concerns regarding thrombi in the pulmonary circulation. Vandam *et al* [28] showed that in COVID-19, thrombotic lesions were more widely distributed in the peripheral arteries of the lung and that total clot burden was lower. Their data suggest that the phenotype of Pulmonary Embolism (PE) in patients with COVID-19 may be different from the PE phenotype in patients without COVID-19 pneumonia. We have all seen cases of arterial and venous thrombus appearing after discharge, or patients returning after discharge for dyspnoea, without interstitial lung disease and with alterations in CO transfer. This is probably due to peripheral “*in situ*” thrombosis of small vessels. In any case, this indication has not been proven to be effective.

### 1.3 Treatment of Hypoxia

To complete the treatment, we should focus now on the therapeutic options for hypoxia. Many of these patients have normal lung function and should, therefore, be treated in a totally different way from chronic respiratory patients who are hypoxic and hypercapnic. In stage I, oxygen supplement should be enough. Since some patients may develop peripheral atelectasis, I would start short sessions of CPAP (two hours in the morning and two in the afternoon at least) to try to prevent this atelectasis, and train the patient in case they deteriorate and need prolonged sessions of CPAP. In stage II, permanent CPAP or Non-Invasive Mechanical Ventilation (NIMV) should be considered. Since the respiratory drive of these patients is high, low values of pressure support may cause high tidal volumes (>1.000 ml). I advise the use of face masks rather than oro-nasal masks, for two reasons: first, to reduce airway leaks, and second, because these patients need to be fed due to the high catabolism of the disease. The presence of a gastric tube itself produces an airway leak and thus puts health staff at a higher risk of exposure. With the normal face mask, this risk is lower. Fig. (**2**) shows the indications for each technique.

When a patient with respiratory failure cannot be adequately oxygenated and requires additional measures, we should decide first whether he is suitable for Intubation (IT) and Invasive Mechanical Ventilation (IMV). If the answer is affirmative, then no time should be wasted: these patients deteriorate abruptly and a delay in intubation may be fatal. Patients not considered suitable for IT may be put on CPAP or NIMV, depending on their tolerance. In some cases High Oxygen Flow (HOF) can be applied, although its use is sometimes discouraged due to the increased risk of viral spread [29]. The patient’s presence in a ventilated room or in a room with negative pressure aspiration should not be a limitation.

In invasively ventilated patients, there is a dissociation between the relatively well-preserved lung mechanics and the severity of the hypoxemia. Curiously, patients with normal lung compliance have a high right-to-left shunt fraction. This again illustrates the unusual situation we are facing. For these patients, two additional options exist: increasing the Positive End Expiratory Pressure (PEEP) and/or moving them into the prone position [30]. Some patients with classic ARDS will improve due to alveoli recruitment, while patients with refractory hypoxemia due to COVID-19 will show some improvement with these techniques due to the redistribution of perfusion in response to pressure and/or gravitational forces [31]. On the basis of the response to an increase in PEEP, patients can be classified in two phenotypes: phenotype “L” (a low proportion of recruitable patients with near-normal compliance); or phenotype “H” (high potential for recruitment and low compliance, no different from classical non-COVID-19 ARDS). In phenotype L patients, PEEP may lead to severe hemodynamic impairment and fluid retention and the prone position may be less efficient, thus creating an unnecessary extra workload in the context of the pandemic.

The final stage is the post-COVID-19 situation. If the information on the active disease is scarce, the world after the infection remains totally uncharted territory. We have observed benign cutaneous lesions appearing after several weeks of PCR negativization, deterioration of well-established lung fibrosis (with the disease acting as a trigger) and unexpectedly fast development of new fibrosis; with regard to coagulation impairment, we have seen the appearance of stroke episodes [32, 33] and patients seeking medical attention three or four weeks after hospital discharge due to the appearance of dyspnoea, in whom the CT scan rules out lung parenchymal disease but CO transfer is impaired. Possibly, anticoagulation should be considered in some of these patients if inflammatory parameters are elevated.

## CONCLUSION

To summarize, the following concepts should be borne in mind. First of all, a correct staging of the disease can help to decide on the most effective and safest pharmacological option. Prescribing a treatment in the inappropriate phase will add no clinical benefits and may instead increase the toxicity of the treatment. At the present time remdesivir seems to be the only treatment recommended for stage I patients, and therefore the only drug recommended for treating viral replication. For the storm mediators phase, TCZ is the drug with the most substantial scientific evidence to be recommended. Limited prescription of systemic corticosteroid at moderate/high doses seem to offer a certain benefit during the storm cytokine phase; burst is not recommended. It seems reasonable to be generous in the initial prescription and maintenance treatment of LMWH. Second, the disease affects mostly normal lungs which present an unexpected behaviour, in any case this behaviour is absolutely different than chronic respiratory patients. The treatment of respiratory failure should always underpin the therapeutic approach. The early stablishing of therapeutic limitations can be helpful. Third and last, pursuing research into intravascular pathology will probably help to clarify some problematic situations. Some of these conclusions are expected to be nuanced in the near future.

## Figures and Tables

**Fig. (2):**
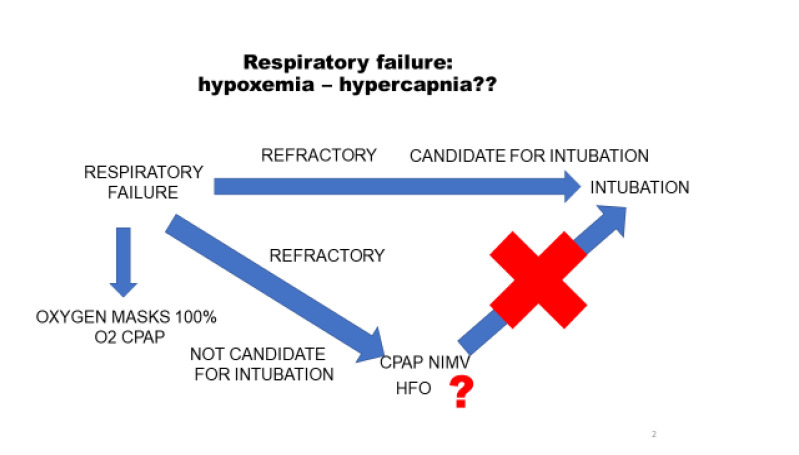
O2: Oxygen. CPAP: Continuous Positive Airway Pressure. NIMV: Non-Invasive Mechanical Ventilation. HFP: High Flow Oxygenation.
